# Seasonal study of the nutritional composition of unexploited and low commercial value fish species from the Portuguese coast

**DOI:** 10.1002/fsn3.2937

**Published:** 2022-05-30

**Authors:** Ana M. Duarte, Frederica Silva, Susana Mendes, Filipa R. Pinto, Sónia Barroso, Elisabete Silva, Ana Neves, Vera Sequeira, Maria Filomena Magalhães, Rui Rebelo, Carlos Assis, Ana Rita Vieira, Leonel Serrano Gordo, Maria Manuel Gil

**Affiliations:** ^1^ MARE – Marine and Environmental Sciences Centre Polytechnic of Leiria Peniche Portugal; ^2^ MARE – Marine and Environmental Sciences Centre, Faculdade de Ciências Universidade de Lisboa Lisbon Portugal; ^3^ MARE – Marine and Environmental Sciences Centre ESTM, Polytechnic of Leiria Peniche Portugal; ^4^ Departamento de Biologia Animal, Faculdade de Ciências Universidade de Lisboa Lisbon Portugal; ^5^ CE3C – Centre for Ecology, Evolution and Environmental Changes Faculdade de Ciências, Universidade de Lisboa Lisbon Portugal

**Keywords:** fish valorization, nutritional composition, seasonal variation, sustainability, unexploited species

## Abstract

Target species diversification is essential for fisheries sustainability and fish market revitalization. Fish discards are a widely recognized problem resulting from fisheries worldwide, and are of major concern for all sector players, from administrations, to fishermen, and scientists. However, non‐target species are seldom studied, and information on nutritional profiles and seasonal changes in nutritional properties is generally lacking. This study assessed the seasonal nutritional composition of two unexploited (*Serranus cabrilla*, *Capros aper*) and three low commercial value fish species (*Trachurus picturatus*, *Spondyliosoma cantharus*, and *Trigla lyra*), captured on the Portuguese coast over 1 year. Significant seasonal variations were observed in the nutritional composition of all the species studied. Moisture and ash contents varied from 70% to 81% and from 5% to 13%, respectively. The maximum fat contents were 5% for *C. aper* and 4% for *T. picturatus*, allowing to classify all studied fishes as lean. The highest protein contents were recorded for *C. aper* (25%) and *S. cantharus* (20%). The unexploited and low commercial value fish species studied were shown to be good fat and protein sources, comparable to commonly consumed species, such as cod and salmon, having a great potential to become commonly consumed fish in Portugal.

## Graphical abstract

Non‐target species are seldom studied and information on nutritional profiles and seasonal changes in nutritional properties is generally lacking. This study assessed the seasonal nutritional composition of two unexploited (*Serranus cabrilla*, *Capros aper*) and three low‐commercial value fish species (*Trachurus picturatus*, *Spondyliosoma cantharus* and *Trigla lyra*), captured off the Portuguese coast over one year. The unexploited and low‐commercial value fish species studied were shown to be good fat and protein sources, having a great potential to become commonly consumed fish in Portugal.

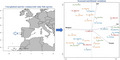

## 1 INTRODUCTION

The growing world population is increasing the nutritional demand, causing the overexploitation of food sources such as fish (FAO, 2020). Thus, discarded fish species could become new alternatives to the consumer's diet, bringing benefits to fishermen as well as to companies with the advantage of limiting the environmental impact. Fish discards may result in the rejection of nutritionally rich seafood, which may have similar contents of bioactive compounds, high‐quality proteins, fats, and minerals, to those found in commonly consumed seafood (Blanco *et al*., 2018).

Fish discards include low or no commercial value species, by‐catches of fish under minimum conservation reference sizes, non‐quota or exhausted quota fish species, and crushed or damaged fish (FAO, 1996). According to the last FAO's update, between 2010 and 2014, the annual discards from global marine capture fisheries were 9.1 million tons, of which 46% were from bottom trawls (Pérez Roda *et al*., 2019). Discarded fish have generally low survival rates leading to consequences in marine ecosystems (Guillen *et al*., 2018). Therefore, the valorization of discarded fish is important not only for the fishery sector but also for marine systems sustainability. In order to avoid and/or reduce fish discards, a landing obligation regulation was implemented in 2013 for the European Fisheries (Regulation [EU] 1380, 2013), which includes all the species under TAC (total allowable catch) regulations.

Low or no commercial value species are commonly used for animal feeding purposes or as raw material for aquaculture feeding. However, 12% of the global fish production fall in this category, demanding alternative solutions for such species (ICES, 2006). One of the approaches to promote the consumption of low or no commercial value species involves the assessment of their nutritional content, given the growing relevance of this topic to consumers and, consequently, to the industry. The main components of fish muscle are water, protein, and lipids, being important constituents when determining its nutritional value. Fish protein has high nutritional value, because it includes all the essential amino acids (e.g., methionine and lysine) and is also highly digestible when compared with terrestrial meat proteins (Tilami & Sampels, 2017). Fish is also an important source of lipids in the human diet, being rich in polyunsaturated fatty acids, such as omega‐3, which contribute to the normal functioning of the cardiovascular, neural, and immunity systems (Tilami & Sampels, 2017). Moisture content is an important measure in food products because it is a quality and safety factor in food preservation (Nielsen, 2010).

**FIGURE 1:**
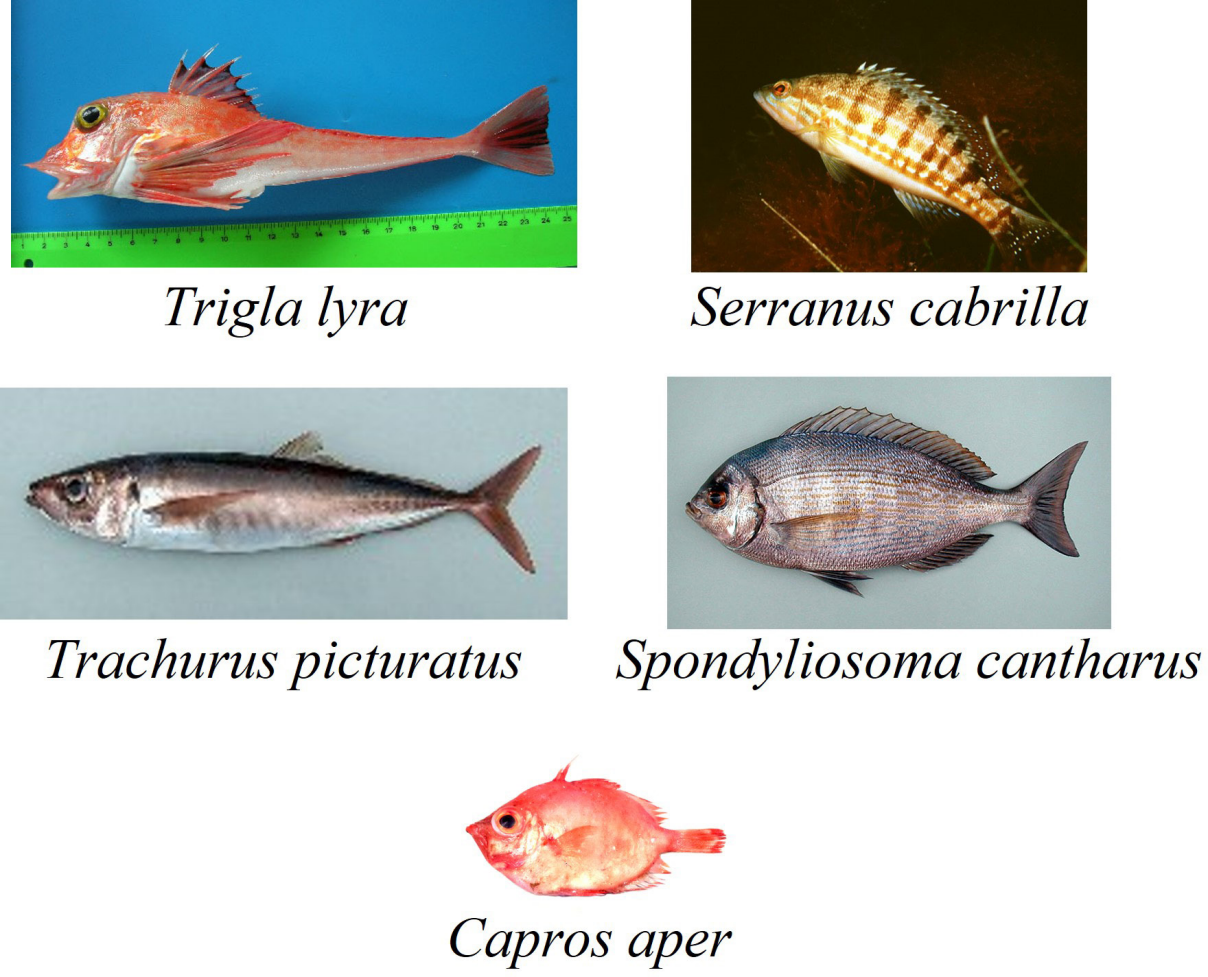
Fish species used in the present study. Source: *T. lyra* (Fishbase, by Ilkyaz, A.T.), *T. picturatus* (Fishbase, by Cambraia Duarte, P. M. N.), *S. cabrilla* (Fishbase, by Hernández‐González, C.L.), *S. cantharus* (Fishbase, by Cambraia Duarte, P. M. N.), *C. aper* (original)

The fish species that are more consumed in Europe are tuna (*Thunnus* sp.), Atlantic cod (*Gadus morhua*, Linnaeus, 1758), and Atlantic salmon (*Salmo salar*, Linnaeus, 1758) (European Commission, 2019). The most consumed fish species in Portugal are tuna, cod, and hake (*Merluccius merluccius*, Linnaeus, 1758) (Barroso *et al*., 2020). However, the most caught fish species on the Portuguese coast are sardines, mackerel, tuna, and horse mackerel (in decreasing order) (INE, 2011–2021). This consumption preference is related to a lack of consumer awareness of other fish species, or due to the apprehensiveness to purchase and cook unfamiliar fish species. Thus, the valorization of unexplored species would be an advantage for the country's economy and sustainability. In this context, five fish species with low or no commercial value captured in the Portuguese coast were selected in order to study their nutritional value (Figure 1). Among the species of low commercial value, blue jack mackerel (*Trachurus picturatus*, Bowdich, 1825), black seabream (*Spondyliosoma cantharus*, Linnaeus, 1758), and piper gurnard (*Trigla lyra*, Linnaeus, 1758) are particularly important for their landings and first auction prices (INE, 2011–2021). Regarding the species without commercial value, comber (*Serranus cabrilla*, Linnaeus, 1758) and boarfish (*Capros aper*, Linnaeus, 1758) are particularly abundant in the Portuguese coast (INE, 2011–2021). The rejection of these fish species by the consumer is mainly a cultural issue, as they are unknown by the majority of the population, despite being savory and nutritious. There are also other factors adding to the low popularity of some of these species, *e.g*., *T. picturatus* is often undervalued due to its darkened muscle coloration in comparison with *Trachurus trachurus*, and *C. aper* is a small fish.

Comber and boarfish are among the most important species in terms of abundance and discards, being mainly caught as by‐catch. *S. cabrilla* belongs to the Serranidae family and can be found in sandy and rocky bottoms at depths up to 500 m in eastern Atlantic from the Bay of Biscay to South Africa and the Mediterranean (Gordo *et al*., 2016). This hermaphroditic species is mainly caught as a by‐catch in trawls and trammel nets and is discarded at sea or grouped in the “diverse” category at Portuguese auctions (Gordo *et al*., 2016; Sequeira *et al*., 2015). *C. aper* belongs to the Caproidae family and is the only species in its genus (Egerton *et al*., 2017). It is found from Norway to Senegal and also in superficial waters of the Mediterranean Sea, but it is more abundant on rocky bottoms at the edge of the continental slopes (Sequeira *et al*., 2015). In general, this species is discarded at sea whenever caught. *T. picturatus* belongs to the Carangidae family, is benthopelagic, and is found in the East Atlantic seas (including Azores Islands, Madeira, and the Canary Archipelagos). Landings on the mainland Portuguese coast, although highly variable, reached an average value of ca 3900 tons/year in the last decade (INE, 2011–2021). In Madeira Archipelago, *T. picturatus* represents about 5% of the total landings, being part of the diet of local population and often used as bait for tuna fish catch (Vasconcelos *et al*., 2006). The Sparidae family, which includes *S. cantharus*, is the most targeted in Portuguese coastal fisheries, being caught by several different fishing gears, with mean annual landings of 220 tons in the last decade (INE, 2011–2021). *S. cantharus* is distributed in the Mediterranean Sea and eastern Atlantic Ocean (including the archipelagos of Madeira, Cape Verde, and Canaries), in rocky and sand substrates up to 300 m deep (Rodríguez *et al*., 2004; Neves *et al*., 2018). *T. lyra* belongs to the Triglidae family and is a demersal fish found in a wide variety of locations, including archipelagos of Azores and Madeira (Papaconstantinou, 1981), with mean annual landings of 370 tons in the last decade (INE, 2011–2021).

The aim of this work was to show that the selected unexploited and low commercial value fish species have a great nutritional potential to be more regularly consumed. Hence, the nutritional composition of the five fish species was analyzed. The study was carried out throughout 1 year since the nutritional parameters may vary along seasons. In previous studies, the selected fish species were shown to be sensorially appealing (Silva *et al*., 2020), and good sources to be used in the development of marine‐based food products with high acceptance from consumers (Silva *et al*., 2021).

## 2 MATERIALS AND METHODS

### 2.1 Sample collection and pretreatment

Fish samples, captured from the coast of Portugal (approximate capture zone between 39°N, 9.5°W and 40°N, 11°W), were acquired at Peniche fish market (center of Portugal) or purchased from local fishermen, every 2 months (Jan/Feb, Mar/Apr, May/Jun, Jul/Aug, Sep/Oct, Nov/Dec), during 2019. Sample preparation was carried out according to Portuguese Standard NP 4424 (2004). Briefly, fishes were washed with deionized type III water, measured for the total length (from mouth to caudal fin, cm), gutted, and filleted, manually rejecting the skin. Then, fish were divided into fresh samples and freeze‐dried samples. To obtain freeze‐dried samples, fish were previously weighted and kept at −80°C for 12 h and then freeze‐dried at −56°C for 60 h (Freeze‐drier Labogene, CoolSafe 55‐4). The freeze‐dried biomass was then crushed using a kitchen robot (Bimby, Vorwerk, Thermomixer 31‐1) and the resulting powder was stored in sampling bags in a fresh, dark, and dry room. Fresh samples were used to determine moisture and ash contents, and dried samples were homogenized to assess total fat and crude protein content. The number of individuals of each species used for the analyses was defined according to their length (NP 4424, 2004). The sample included 10 specimens for individuals up to 15 cm, 5 specimens for individuals up to 30 cm, and for those individuals over 30 cm, 3 cross‐sections with 2.5 cm of each specimen were cut in the area after the pectoral fin, just before the caudal fin and the middle distance between both. Thus, the study included 10 individuals for *S. cantharus*, *T. lyra*, and *T. picturatus*, 20 for *C. aper*, and 15 for *S. cabrilla*, for each period of analysis (for each 2 months).

### 2.2 Nutritional composition analysis

#### 2.2.1 Moisture content

Moisture content assay was performed according to Portuguese Standard NP 282 (1991). Porcelain crucibles were incubated for 3 h at 105 °C, to remove moisture and other substances that could affect the effective crucible weight. After cooling to room temperature in a desiccator, 10 g of fresh sample were weighed into the pre‐weighed crucibles (Analytical Balance Sartorius, ENTIRIS224I‐1S). The crucibles with the samples were placed into a drying oven (Memmert, UF110) at the mentioned temperature and time. The samples were then removed and cooled down to room temperature in a desiccator (ca. 1 h). The samples were kept inside the desiccator and weighed daily until the values from successive weighings did not differ by more than 10 mg (3–4 days). Moisture content was expressed as g/100 g of fresh weight (FW) and calculated using the following equation:

(1) $$\text{Moisture content}=\frac{W_{\text{fresh}}-W_{\text{dehydrated}}}{W_{\text{fresh}}}\times 100$$

where *W*_fresh_ is the fresh sample weigh and *W*_dehydrated_ is the average weigh of the dehydrated sample (average of the three last weighings).

#### 2.2.2 Ash content

The ash content assay was performed according to Portuguese Standard NP 2032 (1988). The crucibles incubation and cooling procedures were the same as described for the moisture assay. For this assay, 5 g of fresh sample were weighed, and the samples were placed in an incinerator (Nabertherm, B170) at 525°C for 4 h. The sample ashes were cooled to room temperature in a desiccator and their weight was recorded until the values from successive weighings did not differ by more than 1 mg. The ash content was expressed as g/100 g of FW and determined using the following equation:

(2) $$\mathrm{Ash}\;\text{content}=\frac{W_{\mathrm{ash}}\;}{W_{\text{fresh}}}\times 100$$

where *W*_ash_ is the average ash weight and *W*_fresh_ is the fresh sample weight.

#### 2.2.3 Total fat content

The total fat determination was adapted from Folch *et al*. (1957). Briefly, 1 g of dried sample was mixed with 0.8 ml of distilled water and 5 ml of Folch reagent and mixed in a vortex (VWR, VV3) for 1 min. Folch reagent was prepared using CHCl_3_ and MeOH (VWR, Fontenay‐sous‐Bois, France) in a proportion of 2:1 (v:v). Further 5 ml of Folch reagent were added and mixed for 5 min, and then for more 2 min after adding 1.2 ml of 0.8% NaCl solution (Biochem, Cosne sur loire, France). The samples were centrifuged (4347 × *g*, 10 min, 4°C; Centrifuge Eppendorf, 5810R) and the lower phase (organic) was filtered through hydrophilic cotton and anhydrous sodium sulfate (VWR, Leuven, Belgium) to round‐bottomed glass flask. To avoid sample loss, more 5 ml of CHCl_3_ were added, followed by a second centrifugation and filtration, under the same conditions. The solvent from the collected organic phases was removed through low‐pressure evaporation using a rotary evaporator (Heidolph, Laborota 4000). After complete solvent evaporation, the samples were placed in an oven at 30°C for 4 h and then kept in a desiccator until a constant weight was registered (less than 10 mg between successive weighings). The total fat content was expressed as g/100 g of FW and determined using the following equation:

(3) $$\text{Total}\;\mathrm{fat}=\frac{W_{\mathrm{fat}}}{W_{\text{sample}}}\times 100$$

where *W*_fat_ is the average weight of the fat (weight of round‐bottomed glass flask with the fat – weight of the empty round‐bottomed glass flask), and *W*_sample_ is the sample's initial weight.

#### 2.2.4 Crude protein content

The total protein assay was performed according to Portuguese Standard NP 4488 (2009), applying the Kjeldahl method. Briefly, 0.5 g of sample (or distilled water in the blank assay) were mixed with 2 Kjeldahl tablets (Panreac) and 25 ml of H_2_SO_4_ (Honeywell, Fluka, MI) in digestion tubes. Samples were further digested in a Kjeldahl digestor (Foss, Digestor2006) at 400°C for 90 min. After samples were cooled down to room temperature, 80 ml of distilled water were added and the ammonia formed was distilled into 30 ml of a 4% H_3_BO_3_ (Panreac) solution containing bromocresol green (Alfa aesar) and methyl red (Panreac), under alkaline conditions (distillation with 40% NaOH, Fisher scientific), using a Kjeldahl distiller (Foss, Kjeltec2100). The distilled samples were titrated with HCl 0.5 M (VWR).

The crude protein content, represented by the sample's nitrogen content, is expressed as g/100 g of FW, and was calculated using the following equation:

(4) $$\text{Protein content}=\frac{6.25\times 0.014\;\left(V_{a}-V_{b}\right)\times N\;}{W}\times 100$$

where 6.25 is the nitrogen‐to‐protein conversion factor (Jones, 1941), *V*_a_ is the volume of HCl spent on sample titration, *V*_b_ is the volume of HCl spent on blank assay, *N* is the HCl normality, and *W* is the sample weight.

### 2.3 Average percentage variation over the year

The variation in the percentage of moisture, fat, and protein over the year was obtained as follows. The variation module between pairs of months was calculated according to the following equation:

(5) $$\text{Variation between pairs of months}\;\left(\%\right)=\frac{\left(M_{i}-M_{i+1}\right)}{\max}\times 100$$

where *M*_i_ is the first pair of months, *M*_i+1_ is the next couple of months, and *max* is the highest value between *M*_i_ and *M*_i+1_. Then, the annual variation was obtained with the average of the variations between pairs of months.

### 2.4 Statistical analysis

One‐way analysis of variance (ANOVA) was performed to identify significant differences in fish length and nutritional composition throughout the year (pairs of months/seasons) for each species. All assumptions required to perform the ANOVA (that is, data normality and homogeneity of variance) were validated (by applying the Shapiro–Wilk and Levene tests, respectively), and the Tukey test was used in post hoc multi‐comparisons (*F value* and *p‐value*) (Zar, 2010). When assumptions were not met, the nonparametric Kruskal–Wallis test and the Games–Howell multi‐comparison test were applied (Zar, 2010). Analyses were performed using IBM SPSS Statistics 27 (IBM SPSS, 2020). Significance of statistical testing was assessed at *p* < .05.

Principal Component Analysis (PCA) (Ter Braak and Smilauer, 2002) was used to summarize patterns in nutritional composition (moisture, ash, total fat, and crude protein contents) among species throughout the months/seasons. PCA model was built using the average of measured data, and full cross‐validation was applied to validate the model. The principal components (PC) were calculated by linear combination of original variables and adequately represent the original data (Bro and Smilde, 2014). The data were mean‐centered by subtracting from every variable the corresponding mean level, and the variables were scaled by dividing them by the corresponding standard deviations. Thus, PCA proved to be adequate, since it allows to reduce the data dimensionality, maintaining relevant information contained therein (Bednárová, 2013). Although only the results concerning the first two components are present, the others were also analyzed. Analyses were performed using Canoco for Windows 4.5 software (Ter Braak and Smilauer, 2002).

All analyses were performed in triplicate. Whenever applicable, the results are presented by the mean ± standard deviation (SD).

## 3 RESULTS AND DISCUSSION

### 3.1 Characterization of the samples

Biotic, abiotic factors or even fishing pressure can influence the growth and consequently the fish length. Among the biotic factors, food availability and low competition for food are the most relevant. Fishing pressure can lead to populations of smaller individuals since the larger ones are more susceptible to be caught by the different fishing gears (King, 2013). According to Webster *et al*. (2011), intraspecific morphological variations can be associated with environmental differences such as flow regime, water depth, water chemistry, and substrate type. Also, the water temperature can influence growth rate, body length, and reproductive schedules, since reduced temperatures result in lower initial growth rate and delayed maturation in larger body length (Trip, 2014).

The total length (cm) and number of specimens used for each species over the year are presented in Table 1. *C. aper* was the smallest species studied, with an annual average length of 12.7 ± 0.6 cm, while *T. lyra* and *T. picturatus* were the longer species, with annual average lengths of about 30 cm. *T. lyra* was also the species that presented the highest annual variation in total length.

**TABLE 1:** Total length (cm) for each species in each pair of months

Species	*n*	Pairs of months						Annual average	Annual variation (%)
		Jan/Feb	Mar/Apr	May/Jun	Jul/Aug	Sep/Oct	Nov/Dec		
*S. cantharus*	10	23.4 ± 0.7^a^	24.5 ± 1.0^ab^	27.2 ± 1.5^cd^	22.2 ± 1.7^a^	29.0 ± 1.0^d^	26.5 ± 2.2^bc^	25.5 ± 2.7^AB^	15 (2–5)
*T. lyra*	20	29.7 ± 1.7^a^	29.4 ± 1.4^a^	27.7 ± 1.4^a^	27.9 ± 1.9^a^	36.4 ± 2.2^b^	29.5 ± 1.4^a^	30.1 ± 3.4^A^	21 (2–42)
*T. picturatus*	15	25.6 ± 1.5^a^	26.7 ± 1.7^a^	40.6 ± 1.2^b^	35.0 ± 1.1^c^	25.7 ± 1.3^a^	26.8 ± 1.0^a^	30.1 ± 5.9^A^	13 (4–23)
*C. aper*	10	12.4 ± 1.6^a^	12.3 ± 0.8^a^	12.9 ± 0.8^a^	12.6 ± 0.9^a^	19.3 ± 1.5^b^	12.7 ± 0.6^a^	13.7 ± 2.7^C^	10 (1–23)
*S. cabrilla*	10	21.8 ± 1.9^a^	21.4 ± 2.2^a^	20.5 ± 1.0^ab^	[#]	11.9 ± 0.9^a^	18.9 ± 2.1^b^	18.9 ± 4.0^BC^	17 (4–34)

*Note:* Values are presented as mean ± SD. Different lowercase letters in rows represent significant differences between pairs of months for each species and different uppercase letters in columns represent significant differences between species (*p*‐value <.05). *n* is the number of specimens used for each pair of months. Annual variation (%) is presented as Av. (Min.–Max.), were Av. is the average of the variations between pairs of months, and Min and Max are the minimum and maximum variations observed between pairs of months, respectively.

[#] Data missing due to mechanical problems in the supply vessel.

The length of *S. cantharus* presented statistically significant differences throughout the year, varying between 22.2 ± 1.7 cm (Jul/Aug) and 29.0 ± 1.0 cm (Sep/Oct) (Table 1). As stated by Neves *et al*. (2018), individuals may be characterized according to total length as juveniles (2.1–13.6 cm), females (9.1–32.2 cm), transition (20.1–32 cm), and males (20.0–38.0 cm). Accordingly, the sample used in this study is mainly composed of mature individuals.

The length of *T. lyra* presented statistically significant differences during the period under analysis, remaining around 27 cm long throughout the year, with a high increase in Sep/Oct, when it reached its maximum length (36.4 ± 2.2 cm) (Table 1). This is in agreement with the results obtained by Sousa (2019) that reported lengths ranging from 12.5 to 45.1 cm for specimens caught in the coast of Portugal for 1 year. According to the author, the total length of the individuals ranged between 12 and 13 cm for immature individuals, between 23.5 and 45.1 cm for females, and between 25.8 and 41.0 cm for males. Taking this information into account, the samples of *T. lyra* used in this study are mainly composed of mature individuals.

*Trachurus picturatus* also presented statistically significant differences in the length throughout the year, with the minimum length (25.6 ± 1.5 cm) in Jan/Feb and the maximum length (40.6 ± 1.2 cm) in May/Jun (Table 1). These values agree with the data reported for *T. picturatus* from Madeira and Azores archipelagos, with lengths ranging from 17 to 46 cm (Vasconcelos *et al*., 2006) and from 15 to 40 cm (Isidro, 1990), respectively.

The average length of *C. aper* through the year also presented statistically significant differences, presenting a constant length throughout the year (about 12 cm) and a maximum value in Sep/Oct (Table 1). These results are in line with what was previously found for this species captured off the Portuguese coast, with a total length ranging between 7.2 and 9.5 cm for immature specimens and 8.1 and 17.0 cm for mature ones (Sequeira *et al*., 2020). A maximum length of 23 cm was reported for *C. aper* from the Northeast Atlantic, but the length range for this species varies between 2.5 and 18 cm with 12 cm being a more commonly observed length (Hüssy *et al*., 2012). Length at maturity was estimated to be at 9.7 cm in this region (Farrel *et al*., 2012), which is similar to the maximum length of an immature fish sampled in the Portuguese coast (9.5 cm).

As for *S. cabrilla* the length also presented statistically significant differences throughout the year, with the lowest length value (11.9 ± 0.9 cm) in Sep/Oct and a constant length of about 20 cm in the other periods (Table 1). These results are similar to those reported for this species captured off the Portuguese coast between July 2011 and September 2012, with a length variation between 12 and 26 cm (Gordo *et al*., 2016).

### 3.2 Nutritional composition

Table 2 presents a summary of the nutritional parameters analyzed every 2 months during 1 year for the five species studied. For all the species, moisture was the highest component (69–82%), followed by protein (14–25%), ash (5–13%), and fat (0.6–5%).

**TABLE 2:** Summary of nutritional analysis for each species in each pair of months

Parameter	Species	Pairs of months						Annual average	Annual variation (%)
		Jan/Feb	Mar/Apr	May/Jun	Jul/Aug	Sep/Oct	Nov/Dec		
Moisture	*S. cantharus*	78.17 ± 0.65^a^	75.86 ± 1.27^ab^	81.57 ± 1.45^c^	72.52 ± 0.87^bd^	72.49 ± 0.50^d^	75.28 ± 0.90^ad^	75.98 ± 3.43^AB^	5 (0–11)
	*T. lyra*	78.66 ± 0.11^a^	71.86 ± 1.71^b^	73.21 ± 0.94^bc^	76.79 ± 0.41^ac^	76.81 ± 0.39^ac^	73.79 ± 1.10^bc^	75.19 ± 2.64^AB^	4 (0–9)
	*T. picturatus*	76.26 ± 2.40^a^	72.32 ± 1.59^ab^	69.46 ± 1.46^b^	70.45 ± 0.72^b^	70.02 ± 0.89^b^	70.67 ± 0.96^b^	71.53 ± 2.79^B^	2 (1–5)
	*C. aper*	74.33 ± 1.79^a^	73.24 ± 0.73^a^	71.43 ± 2.12^a^	73.63 ± 0.85^a^	75.81 ± 1.68^ab^	79.55 ± 0.71^b^	74.66 ± 3.00^AB^	3 (2–5)
	*S. cabrilla*	78.89 ± 1.26^ac^	80.48 ± 1.12^a^	75.30 ± 0.38^b^	[#]	77.30 ± 0.39^bc^	78.11 ± 0.18^ac^	78.02 ± 1.96^A^	3 (1–6)
Ash	*S. cantharus*	11.37 ± 0.17^a^	12.95 ± 0.10^b^	12.06 ± 0.35^a^	9.55 ± 0.13^c^	10.03 ± 0.22^c^	11.36 ± 0.38^a^	11.22 ± 1.21^A^	11 (5–12)
	*T. lyra*	6.45 ± 0.14^ab^	6.77 ± 0.60^ab^	8.77 ± 0.47^a^	5.84 ± 0.20^b^	6.03 ± 0.15^b^	7.11 ± 0.28^a^	6.83 ± 1.06^B^	16 (3–33)
	*T. picturatus*	5.23 ± 0.58^a^	7.13 ± 0.47^b^	7.36 ± 0.52^bc^	7.56 ± 0.31^bd^	8.65 ± 0.24^cd^	8.91 ± 0.48^d^	7.47 ± 1.32^B^	10 (3–27)
	*C. aper*	8.09 ± 0.11^a^	10.11 ± 0.34^b^	10.34 ± 0.50^b^	10.67 ± 0.30^b^	11.97 ± 0.32^c^	12.86 ± 0.23^c^	10.67 ± 1.58^A^	9 (2–20)
	*S. cabrilla*	6.83 ± 0.29^a^	5.96 ± 0.20^a^	6.43 ± 0.45^a^	[#]	6.75 ± 0.16^a^	6.25 ± 0.21^a^	6.45 ± 0.44^B^	8 (5–13)
Total fat	*S. cantharus*	2.19 ± 0.13^a^	1.17 ± 0.06^b^	0.98 ± 0.06^b^	2.11 ± 0.17^a^	2.42 ± 0.28^a^	2.28 ± 0.19^a^	1.86 ± 0.60^AB^	27 (6–53)
	*T. lyra*	1.65 ± 0.10^a^	1.21 ± 0.09^b^	0.88 ± 0.06^cd^	0.79 ± 0.06^c^	1.14 ± 0.07^bd^	2.02 ± 0.09^e^	1.28 ± 0.60^BC^	28 (10–43)
	*T. picturatus*	3.47 ± 0.32^a^	2.00 ± 0.05^b^	4.40 ± 0.14^c^	3.68 ± 0.10^ad^	4.33 ± 0.32^cd^	4.41 ± 0.19^c^	3.71 ± 0.90^A^	26 (2–54)
	*C. aper*	1.68 ± 0.09^a^	1.01 ± 0.15^b^	5.01 ± 0.19^c^	3.43 ± 0.16^d^	1.86 ± 0.09^a^	1.52 ± 0.05^a^	2.42 ± 1.42^AB^	43 (18–80)
	*S. cabrilla*	0.66 ± 0.08^a^	0.74 ± 0.01^a^	0.74 ± 0.10^a^	[#]	0.60 ± 0.01^a^	0.65 ± 0.01^a^	0.68 ± 0.09^C^	10 (1–20)
Crude protein	*S. cantharus*	17.31 ± 0.19^a^	16.28 ± 0.30^b^	16.09 ± 0.12^b^	16.51 ± 0.16^ab^	20.02 ± 0.21^c^	18.27 ± 0.39^d^	17.41 ± 1.44	7 (1–18)
	*T. lyra*	17.20 ± 0.07^ab^	18.35 ± 0.19^c^	17.44 ± 0.11^a^	16.80 ± 0.09^b^	19.30 ± 0.22^d^	19.55 ± 0.08^d^	18.11 ± 1.08	6 (1–13)
	*T. picturatus*	17.43 ± 0.10^a^	17.60 ± 0.17^ab^	17.87 ± 0.13^b^	18.82 ± 0.03^c^	18.52 ± 0.17^c^	18.46 ± 0.08^c^	18.12 ± 0.54	2 (0–5)
	*C. aper*	11.69 ± 0.27^a^	15.62 ± 0.12^b^	17.89 ± 0.61^c^	24.91 ± 0.36^d^	15.68 ± 0.07^b^	14.49 ± 0.18^e^	16.71 ± 4.24	22 (8–37)
	*S. cabrilla*	15.17 ± 0.21^a^	17.16 ± 0.04^b^	17.86 ± 0.12^c^	[#]	17.08 ± 0.16^b^	17.76 ± 0.14^c^	17.01 ± 1.01	6 (4–12)

*Note:* Values are presented as mean ± SD, and expressed as g/100 g of fresh weight. Different lowercase letters represent significant differences between pairs of months for each species and different uppercase letters in columns represent significant differences between species for each parameter (*p*‐value <.05). Annual variation (%) is the average of the variation between pairs of months.

[#] Data missing due to mechanical problems in the supply vessel.

#### 3.2.1 Moisture content

Moisture content varied throughout the year for *S. cantharus*, *T. lyra*, *T. picturatus*, *C. aper*, and *S. cabrilla* (Table 2). Despite these variations, moisture content did not present a wide variation throughout the year for the species studied, with moisture values ranging between 69% and 82% (Table 2). The moisture content of raw fish is reported to be around 79% [10], which is in line with the average moisture content obtained for the species under study.

*Spondyliosoma cantharus* moisture content in May/Jun was significantly higher than observed for other months, decreasing until Sep/Oct, when it reached its minimum (Table 2). Considering other species from *S. cantharus* family, there is some similarity between moisture contents, such as black seabream (*Pomadasys peroteti*) from Gabon with 78.50 ± 0.14% (Ondo‐Azi *et al*., 2013). However, higher contents were reported for *Sparus aurata* captured in April in the Central Adriatic (79.12 ± 0.48% on wet weight basis) (Šimat *et al*., 2012). In addition, seabream (*Argyrops spinifer*) captured in Oman, revealed different seasonal moisture contents, with the maximum content being reported in September (78.6 ± 0.3 g/100 g of fish flesh) and the minimum in June (77.8 ± 0.7 g/100 g of fish flesh) (Ali *et al*., 2013).

The average moisture content of *T. lyra* was similar to that reported in a study by Blanco *et al*. (2018) on *Trigla* spp. captured off the northwestern coast of the Iberian Peninsula (74.320 ± 0.530%). Similar results were reported for tub gurnard (*Chelidonichthys lucernus*), which belongs to the Triglidae family, captured off the north‐eastern Mediterranean coast of Turkey in January (77.79 ± 0.40%) and October (75.95 ± 0.15%) (Küçükgülmez *et al*., 2010). However, the study reported higher moisture content in specimens captured in April (78.34 ± 0.12%), compared with the results obtained in the present study at the same time of the year for *T. lyra*.

The average moisture content obtained for *T. picturatus* was similar to the value reported for *T. picturatus* captured in north‐eastern Atlantic (74.970% ± 1.630 in ground tissue wet weight) (Nogueira *et al*., 2013), and with those reported for horse mackerel (*T. trachurus*) captured in Nigeria, with 73.56 ± 0.01% of moisture content (Adeyemi *et al*., 2013). However, higher contents were reported for *Trachurus murphyi* from Chile with a moisture content of 75.37 ± 0.91 g/100 g (Bastías *et al*., 2017).

Regarding *C. aper*, the average moisture content obtained is similar to the value reported by Spitz *et al*. (2010) of 71.3% of wet total body mass, captured in the Bay of Biscay in autumn from 2002 to 2008, but the moisture content obtained in Nov/Dec (autumn) in the present study was higher.

The average moisture content of *S. cabrilla* is comparable to that reported by Edirisinghe *et al*. (2013) for *Epinephelus merra* male specimens from the Serranidae family, captured in Sri Lanka, with 77.17 ± 0.38 g/100 g fresh wet mass and higher than female individuals (74.31 ± 1.78 g/100 g fresh wet mass).

#### 3.2.2 Ash content

The ash content refers to the inorganic residue remaining after incineration of fresh sample, representing the total mineral content in foods (Nielsen, 2010). Ash content by species and months is presented in Table 2. The variation in ash content throughout the year was statistically significant for *S. cantharus*, *T. lyra*, *T. picturatus*, and *C. aper*. The highest average variation in ash content throughout the year was observed for *T. lyra* (16%), while for the other species the variation was in average 8–11%. Ash content ranged between 5% and 13% for the species and period studied, which is higher than the ash content of 2.5% reported for raw fish on a wet weight basis (Nielsen, 2010). However, the values obtained are in agreement with what is reported in the literature, including ash content ranges from 0.7 to 5.3% (Bogard *et al*., 2015) and from 1.9 to 17.8% (Flowra *et al*., 2012).

*Spondyliosoma cantharus* average ash content was noticeably higher when compared with other species from the Sparidae family. For example, *P. peroteti* from Gabon presented an ash content of 1.01 ± 0.14% (Ondo‐Azi *et al*., 2013) and *S. aurata* captured in April in the Central Adriatic Sea had an ash content of 1.12 ± 0.06% (Šimat *et al*., 2012). Kiczorowska *et al*. (2019) also reported a lower ash content for *S. aurata* captured in the Mediterranean Sea (1.37 ± 0.02 g/100 g of fish). The ash content found for *T. lyra* average was higher than the ash contents reported for species from Triglidae family, such as *C. lucernus* captured in Nigeria in January (1.38 ± 0.04%), April (1.35 ± 0.01%), and October (1.34 ± 0.01%) (Küçükgülmez *et al*., 2010), and *C. lucerna* captured in the Baltic Sea (0.99 ± 0.03 g/kg of fish) (Kiczorowska *et al*., 2019). *T. picturatus* average ash content was similar to that reported by Adeyemi *et al*. (2013) for *T. trachurus* captured in Nigeria with 8.60 ± 0.20% of ash. However, lower contents were reported for *T. murphyi* from Chile, with an ash content of 0.90 ± 0.09 g/100 g (Bastías *et al*., 2017). The average ash content obtained for *C. aper* in the present study is higher than the value reported by Spitz *et al*. (2010) with 4.6% of wet total body mass, captured in the Bay of Biscay in autumn. The average ash content obtained for *S. cabrilla* was similar to that reported by Edirisinghe *et al*. (2013) for *E. merra* male and female specimens, captured in Sri Lanka, with 5.67 ± 0.88 and 5.36 ± 0.56 g/100 g dry mass, respectively.

The obtained range of ash content indicates that the species studied are a good source of minerals. However, the average ash content obtained for all the species is significantly higher than most of those reported in the literature. A possible explanation for this may have been the accidental inclusion of bones as edible parts in the manual filleting, which would have resulted on higher ash content in the samples.

#### 3.2.3 Fat content

Variations in the fat content among pairs of months were statistically significant for *C. aper*, *S. cantharus*, *T. lyra*, and *T. picturatus*, but not for *S. cabrilla* (Table 2). Fat content was the parameter with the higher annual variations in the species studied, with annual variations ranging from 10% (*S. cabrilla*) to 43% (*C. aper*). A high annual variation in the lipid content of fish is usual, being mostly related to the life cycle and energy intake of fish (Taşbozan & Gökçe, 2017).

The fat content obtained for *S. cantharus* decreased throughout the winter (Jan/Feb and Mar/Apr), reaching its minimum value by the spring (May/Jun), followed by an increase until the late summer (Sep/Oct), when it reached its maximum value. Values in the same range but for different seasons were reported for *Sparidae* species, such as *A*. spinifer captured in Oman, which presented a fat content of 0.66 ± 0.1 g/100 g and 4.19 ± 1.2 g/100 g of fish flesh in September and June, respectively (Ali *et al*., 2013). Lower fat values were reported for *P. peroteti* from Gabon (0.67 ± 0.03%) (Ondo‐Azi *et al*., 2013) and *S. aurata* captured in April in the Central Adriatic (0.86 ± 0.12%) (Šimat *et al*., 2012). The fat content found for *T. lyra* was lowest in Jul/Aug with no statistical differences with May/Jun, whereas the maximum fat content was observed in Nov/Dec. Similar fat contents were reported for *C. lucernus* from Nigeria, with a maximum fat content observed in October (3.08 ± 0.04%) and minimum in April (0.26 ± 0.02%) (Küçükgülmez *et al*., 2010). *T. picturatus* fat content was statistically similar for all the months except for Mar/Apr. The minimum fat content was obtained in Mar/Apr, while the maximum value was obtained in Sep/Oct. Similar fat content was reported for *T. trachurus* captured in Nigeria with 3.32 ± 0.01% fat content (Adeyemi *et al*., 2013), but lower values were reported for *T. murphyi* from Chile with a fat content of 6.37 ± 0.21 g/100 g (Bastías *et al*., 2017). The species with the widest variation in fat content along the year was *C. aper*, with a maximum value in Jul/Aug. This could be related to an increase in food availability in the west coast of Portugal where strongest summer upwelling occurs (July to September) (Fiúza *et al*., 1982). Spitz *et al*. (2010) reported a fat content of 4.8% of wet total body mass for *C. aper* captured in the Bay of Biscay in autumn, which is higher than the value obtained for Nov/Dec in the present study (1.52%, Table 2), but similar to the maximum fat content of 5% obtained in May/Jun in this study. These variations may be due to the different environmental conditions and food availability in the two locations along the year. *S. cabrilla* had the lowest fat content among the fish species studied, with an average fat content of 0.68 ± 0.09 g/100 g and no statistical differences throughout the year (Table 2).

According to their fat content, fish may be classified as lean fish (less than 5% fat), such as cod, mid‐fat fish (5–10% fat), such as bream, and fatty fish (10–25% fat), such as some salmon species, although the classification and the lipid ranges in fish may vary among publications (Taşbozan & Gökçe, 2017). The species studied herein presented average fat contents ranging between 0.7% and 3.7% and may be considered lean fishes according to the referred classification. Additionally, the fat contents obtained for the species in the present study are comparable to those reported for commonly consumed fish such as *G. morhua*, with a fat content of 0.1–0.9% (Murray & Burt, 1991), and *M. merluccius*, with a fat content of 0.27–1.24% (Küçükgülmez *et al*., 2008), and inferior to fatty fish, such as *Thunnus* sp., with 25% of fat (Murray & Burt, 1991).

#### 3.2.4 Protein content

Variations in the protein content among pairs of months were statistically significant for *S. cantharus*, *T. lyra*, *T. picturatus*, *C. aper*, and *S. cabrilla* (Table 2). *C. aper* was the species that presented the widest variation in protein content throughout the year (22% average variation), while *T. picturatus* was the one that presented the smallest variation of protein in its composition throughout the year (2%).

The protein content found for *S. cantharus* was remarkably different in Sep/Oct, presenting the highest value of 20%, while the minimum value was observed in May/Jun. Similar protein contents were reported for *S. aurata*, from Sparidae family, captured in the Mediterranean sea, with a protein content of 21.69 ± 0.02 g/100 g of fish (Kiczorowska *et al*., 2019), and for *S. aurata* captured in April in the Central Adriatic Sea, with a protein content of 19.87 ± 0.36% on wet weight basis (Šimat *et al*., 2012). *T. lyra* protein content was remarkably different in Mar/Apr (late winter) when compared to the other months (Table 2). The maximum protein values were obtained in Nov/Dec, while the minimum was observed in Jul/Aug. The obtained protein values are in agreement with those reported by Blanco *et al*., for *Trigla* spp. captured off the northwestern coast of the Iberian Peninsula (Blanco *et al*., 2018). The authors reported a protein content of 17.270 ± 0.140%, which is similar to the average value obtained in the present study throughout the year (18%, Table 2). *T. picturatus* revealed a minimum protein content in Jan/Feb, and a maximum value in Jul/Aug. Similar values were reported for *T. murphyi* from Chile, with a protein content of 16.58 ± 0.88 g/100 g (Bastías *et al*., 2017), but a much higher protein content was reported for *T. trachurus* captured in Nigeria (65.65 ± 1.01%) (Adeyemi *et al*., 2013). The minimum protein content of *C. aper* was observed in Jan/Feb, reaching its maximum value in Jul/Aug. Lower values were reported for *C. aper* captured off the northwestern coast of Ireland with an average protein content of 6.1 ± 1.0% (Egerton *et al*., 2020). However, comparable protein contents (17.2%) were reported for *C. aper* captured in the Bay of Biscay in autumn (Spitz *et al*., 2010). The wide variations in protein content observed throughout the year for this species, with a maximum value in Jul/Aug, could be related to an increase in food availability in the western coast of Portugal in that period (Fiúza *et al*., 1982; Mittelstaedt, 1986), similar to what was observed for the variation of its fat content along the year. *S. cabrilla* protein content was minimum in Jan/Feb, reaching its maximum value in May/Jun, which was not statistically different from the value obtained in Nov/Dec.

Fish protein content is usually between 15 and 20%, although some species may present protein contents out of this range (Murray & Burt, 1991). The protein contents obtained for the species in the present study (14–25%) are within the aforementioned protein contents. Moreover, the protein contents obtained are comparable to those reported for commonly consumed fish, such as *G. morhua* with 15% of protein (Murray & Burt, 1991), *S. salar* with 19–21% (Costa *et al*., 2015), *M. merluccius* with 18–21% (Küçükgülmez *et al*., 2008), and *Thunnus* sp. with 25% of protein (Murray & Burt, 1991). Therefore, these unexploited and low commercial value fish species are a good protein alternative to the commonly consumed fish species.

### 3.3 Seasonal variations in the nutritional composition

**FIGURE 2:**
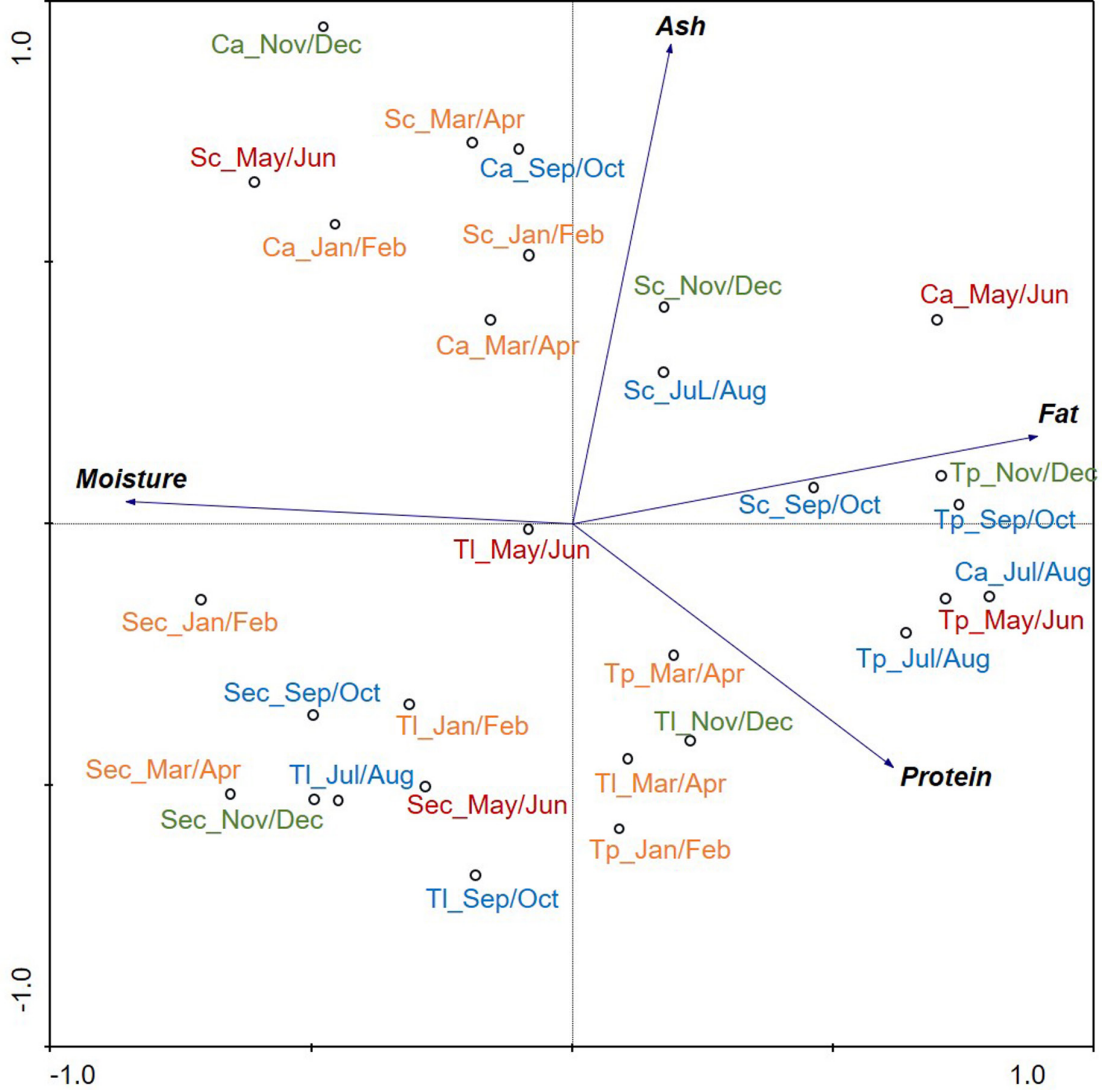
Biplot of the first two principal components representing variation in moisture, fat, protein, and ash contents of each species under study over the months/seasons. PC1 and PC2 explain 48.5% and 27.2% of data variability, respectively. Orange letters – winter (Jan/Feb and Mar/Apr); red letters – spring (May/Jun); blue letters – summer (Jul/Aug and Sep/Oct); green letters – autumn (Nov/Dec); Sc: *S. cantharus*; Tl: *T. lyra*; Tp: *T. picturatus*; Ca: *C. aper*; Sec: *S. cabrilla*

A PCA was conducted to determine possible variations in the nutritional composition of the sudied species (Figure 2). Since the analyses were performed every two months, the following association of months with seasons was considered: winter – Jan/Feb and Mar/Apr; spring – May/Jun; summer – Jul/Aug and Sep/Oct; autumn (Nov/Dec). The first component (PC1) is characterized by the opposition between moisture and fat, as well as by the opposition of moisture with ash and protein (although with less contribution) explaining 48.5% of nutritional data variability. PC2 is characterized by the opposition between ash and protein and explains 27.2% of nutritional data variability. The two main components (PC1 and PC2) together explain 75.7% of the total data variability. PC1 indicates that higher moisture levels are associated with lower values of fat, protein, and ash, while PC2 indicates that higher levels of ash are associated with lower levels of protein (these correlations will be discussed further in the next paragraphs).

*Trachurus picturatus* captured in summer/autumn presented higher fat and protein levels. The seasonal variations on the fat content of *T. picturatus* could be explained by its spawning period. The reproductive season of *T. picturatus* occurs in winter–spring, with the reproductive period starting in December and extending until May, with a peak in February/March (Garcia *et al*., 2015; Vasconcelos *et al*., 2017). The minimum protein and fat levels are observed in winter, during the spawning season. In this period, both fat and protein are used to fulfill the higher energy demands. During breeding, the lipid fraction is the nutritional component with the widest variation, reaching its minimum value around that period. This occurs because the spawning requires higher energy intake, so the energy reserves are spent and most of the species do not ingest much food during spawning migration, enabling the intake of energy through feeding (Huss, 1995).

Higher levels of fat are also associated with *C. aper* in spring (May/Jun) and higher protein contents are associated with *C. aper* in summer (Jul/Aug). This species spawns all year round off the Portuguese coast, with a spawning peak from June to August (Sequeira *et al*., 2015). The spawning period for *C. aper* does not correspond to the period of the year when the lower fat and protein contents are observed, contrasting with what was observed for *T. picturatus*. The seasonal variations in protein and fat contents for this species could be related to an increase in food availability in the west coast of Portugal in that period (Fiúza *et al*., 1982; Mittelstaedt, 1986).

Regarding *T. lyra*, higher protein and fat levels are observed in autumn (Nov/Dec). This species has a short spawning season, from November to February with a peak in January (Sousa, 2019). Thus, the maximum fat and protein contents coincided with the beginning of the spawning season, while minimum levels of these components were observed in the summer.

As for *S. cantharus*, higher moisture levels were observed in the spring (May/Jun). In general, it was observed that muscle moisture content varied inversely with the amount of fat (Ali *et al*., 2020). The increase in fat content and the simultaneous decrease in water content may be related with an increase in age (increasing size). The fluctuations in water, fat, protein, and ash contents with the age may also be related to migration and spawning. This was clearly observed for *S. cantharus* that presents maximum moisture levels in spring when the fat and protein contents are minimum. This corresponded to the end of the spawning season of this species, which usually occurs between February and May (Neves *et al*., 2018).

Regarding *S. cabrilla*, this was the species that presented the smallest annual variation in the nutritional parameters studied (Table 2). As shown in Figure 2, no strong associations could be observed between any of the nutritional parameters and the months/seasons analyzed. Despite of this, the maximum protein and fat contents for this species were obtained in spring, which is coincident with the beginning of the spawning season of this species in the Portuguese coast that occurs from May to July (Sequeira *et al*., 2015).

## 4 CONCLUSION

This study assessed the seasonal nutritional composition of unexploited or low commercial value fish species captured on the Portuguese coast over one year. The species studied were mainly composed by water (69–82%), protein (14–25%), ash (5–13%), and fat (0.6–5%). Significant seasonal variations were observed in the nutritional composition of all the species studied. Fat was the parameter that presented the widest variations throughout the year, with notable differences in *C. aper* from different months. All the studied fish species were considered lean, according to their fat content. *C. aper* and *S. cantharus* were found to have high protein contents. The fish species studied were shown to have high fat and protein contents, comparable to those of commonly consumed species such as *G. morhua* and *M. merluccius*. Therefore, these species demonstrate a great potential to become more commonly consumed fish species, either for human or animal consumption.

The observed seasonal differences appeared to be related to the reproductive cycle of the species, but many other environmental factors, such as food availability, temperature, age, size, and sex, may also contribute to these variations. *C. aper* presented higher fat and protein contents in July/August, while for the other species these parameters were higher from September to December, except for the fat content of *S. cabrilla*, which remains constant throughout the year. For industrial applications, the seasonal variations regarding either the landings of the species or their nutritional composition could be a limitation and should be taken into account.
